# Ensuring Assisted Living Provides the Assistance Residents Need

**DOI:** 10.1001/jamanetworkopen.2022.33877

**Published:** 2022-09-01

**Authors:** Kenneth Lam, Kenneth E. Covinsky

**Affiliations:** Division of Geriatrics, Department of Medicine, University of California, San Francisco

In *JAMA Network Open*, Zimmerman et al^1^ present the results of their Delphi panel on assisted living (AL). They asked health care professionals and elder care experts: if you had to choose based on benefit to quality of life and feasibility, what medical and mental health care ought to be provided in AL facilities?

Interpreting their findings first requires an understanding of what AL is and is not. Many people move into ALs to get assistance as they get older. But an AL facility is not a nursing home (NH). Though the two may be easily confused, NHs are comprehensively accountable for almost all aspects of care for its residents, with regulations around residents’ rights, quality of care, due process for complaints, and transparency of ownership, among other things.^2^ Federal and state regulations must be met for NHs to receive Medicare and Medicaid funding. Residents are chronically and often severely ill, usually from some health condition requiring daily help from a nurse or substantial help with activities like bathing, dressing, and/or eating.^3^ Most NH residents also meet financial criteria to have their care covered by Medicaid.^4^

By contrast, ALs are loosely regulated, which makes defining them and what happens in them challenging. The sector has many synonyms (board and care facilities, group homes, residential care facility), and is defined by its heterogeneity; AL is many institutions of different sizes providing different levels of care. The AL market thus mostly consists of individual contracts for care made between private clients or their representative and a variety of AL businesses. They were originally developed to fill gaps in care for persons before they needed an NH, but are increasingly an alternative to NH. AL regulations occur at the state level. Most residents pay privately.^5,6^

In an ideal world, the less regulated private market for AL would match the demands of their residents with their willingness to pay. Someone looking for help only with bathing on Monday, Wednesday, and Friday would be able to find a place providing that and pay for it, saving money on unneeded services. The provided services, how they benefit a person, and the costs would be upfront and clear. If they were not what was wanted, customers could take their business elsewhere.

The problem is that prospective AL clients often want assistance without granular knowledge of what that assistance is, should, or could be. They also often want that assistance urgently and for things they have never needed assistance with before. They often do not have the time to methodically consider all options nor the experience to appraise them. People first think of and learn about these facilities (and adjacent options such as home care, respite, adult day centers, senior’s homes, and retirement homes) as they become vulnerable from impairments due to some new medical condition or accumulation of old ones. Staying home any longer risks injury. Dizzying and arbitrary definitions about what is or is not assisted living and what is or is not skilled care or medical care are not front of mind. Yet prospective AL clients, by virtue of the mostly unregulated system governing ALs, are left responsible for advocating for themselves in negotiations as they simultaneously come to terms with unfamiliar life circumstances. The ALs, as private, profit-seeking businesses that serve shareholders, have every advantage in the negotiation, and every incentive to bring in customers, upsell services, and provide care as economically as possible; each resident is worth upwards of $50 000 a year. They do not have an incentive to right-size the care to the needs of the client and offer alternatives if the fit is wrong. Residents have little recourse if the AL fails to provide enough staff to deliver promised services.

This is the context for the Delphi panel created by Zimmerman et al. The market being what it is, and its customers being who they are, what obligations should ALs have to their clients to ensure medical and mental health issues are cared for? Prior work by Zimmerman and colleagues^7^ has already shown that ALs can no longer say they care for a mostly well population that can advocate for itself: AL residents today are older, more medically complex, cognitively impaired, and functionally disabled than before.

The panel made 43 recommendations. Taken together, the recommendations emphasize that ALs should be responsible for caring for cognitive decline and mental health needs, and that the best way to care for these issues is personalizing the care rather than providing more medical services. The panel recommended focusing on establishing person-centered care plans; improving training, strategies, and policies for dementia, mental health, and behaviors; increasing staffing; and improving integration of information and care with the medical system. Toenail care and reducing antipsychotics were rated of greater medical and mental health importance than making x-rays, bloodwork, and intravenous fluids available.

The panel thus agreed that ALs should be held accountable for claiming expertise in the care of older persons and common problems that come with aging. We agree. We have seen places where a memory care unit charges upwards of $10 000 a month for “dementia care,” yet is little more than a locked door to prevent residents from leaving the unit and not the sensitive and personalized care advertised.

Moving forward, the next question is how recommendations can be implemented in a heterogeneous sector. We worry a list of 43 items may be too broad for rapid adoption. Interventions to improve AL care will require equipping residents and their representatives to make better decisions about their options; they ultimately have the greatest stake in ensuring high-quality care in a mostly consumer-driven market. The results from this panel could be streamlined into specific questions to guide conversations between prospective residents and ALs to better understand how needs will be met as circumstances change. Questions like “how do you develop care plans for your residents?” and “how do you manage aggressive behaviors?” would help families envision potential challenges that professionals know arise but which the families may not. Prospective residents and caregivers can then decide for themselves if the fit is right and worth the price.

Still, much more must be done to ensure residents are informed consumers of AL and to keep ALs accountable for providing compassionate and dignified care. Research should ask those with experience living in ALs what protections they wish were in place–or at least, what advice current residents might offer prospective ones. It should also ask high-performing ALs and their frontline staff what complaints are unreasonable; we are aware that sometimes, staff carry the burden of impossible expectations handed to them and that sentiment toward ALs has soured since the COVID-19 pandemic. We suspect such research will unearth many more issues: the need for transparent pricing for a la carte options, clear policies for transfer to higher levels of care such as NHs, and clear pathways to investigate allegations of neglect and elder abuse. Without defined standards, it is much easier to take advantage of older persons and their families going through a difficult phase of life than it is to supply high-quality care, and impossible to reward the ALs and paid caregivers that do good work.
